# EGFR and Prion protein promote signaling via FOXO3a‐KLF5 resulting in clinical resistance to platinum agents in colorectal cancer

**DOI:** 10.1002/1878-0261.12411

**Published:** 2019-02-08

**Authors:** Caroline J. Atkinson, Futoshi Kawamata, Cheng Liu, Sunyoung Ham, Balázs Győrffy, Alan L. Munn, Ming Q. Wei, Andreas Möller, Vicki Whitehall, Adrian P. Wiegmans

**Affiliations:** ^1^ Tumour Microenvironment Lab QIMR Berghofer Medical Research Institute Herston Australia; ^2^ Menzies Health Institute Queensland and School of Medical Science Griffith University Southport Australia; ^3^ Department of Gastroenterological Surgery Hokkaido University Graduate School of Medicine Sapporo Japan; ^4^ Conjoint Gastroenterology Laboratory QIMR Berghofer Medical Research Institute Herston Australia; ^5^ MTA TTK Lendület Cancer Biomarker Research Group Hungarian Academy of Sciences Budapest Hungary; ^6^ 2nd Department of Pediatrics Semmelweis University Budapest Hungary

**Keywords:** cisplatin, colorectal cancer, FOXO3a, Prion protein, signal transduction

## Abstract

Epidermal growth factor receptor (EGFR) supports colorectal cancer progression via oncogenic signaling. Anti‐EGFR therapy is being investigated as a clinical option for colorectal cancer, and an observed interaction between EGFR and Prion protein has been detected in neuronal cells. We hypothesized that PrP^C^ expression levels may regulate EGFR signaling and that detailed understanding of this signaling pathway may enable identification of resistance mechanisms and new actionable targets in colorectal cancer. We performed molecular pathway analysis following knockdown of PrP^C^ or inhibition of EGFR signaling via gefitinib to identify changes in expression of key signaling proteins that determine cellular sensitivity or resistance to cisplatin. Expression of these proteins was examined in matched primary and metastatic patient samples and was correlated for resistance to therapy and progression of disease. Utilizing three colorectal cancer cell lines, we observed a correlation between high expression of PrP^C^ and resistance to cisplatin. Investigation of molecular signaling in a resistant cell line revealed that PrP^C^ contributed to signaling via colocalization with EGFR, which could be overcome by targeting p38 mitogen‐activated protein kinases (p38 MAPK). We revealed that the level of Krüppel‐like factor 5 (KLF5), a target downstream of p38 MAPK, was predictive for cell line and patient response to platinum agents. Further, high KLF5 expression was observed in *BRAF*‐mutant colorectal cancer. Our study indicates that the EGFR to KLF5 pathway is predictive of patient progression on platinum‐based therapy.

**Abbreviations**
EGFR: epidermal growth factor receptor
FOLFOX: fluorouracil, leucovorin plus oxaliplatin
FOXO: forkhead/winged helix box class O
KLF5: Krüppel‐like factor 5
p38 MAPK: p38 mitogen‐activated protein kinases
PI3K: phosphoinositide 3‐kinase
PrP^C^: cellular Prion protein

## 1 Introduction

Platinum‐based therapy is frontline for colorectal cancer. The use of fluorouracil, leucovorin plus oxaliplatin (FOLFOX) has seen advances in overall clinical response for aggressive colorectal cancer, with patient survival extended beyond 2 years (Fuchs *et al*., 2007). However, chemoresistance, clinical relapse, and metastasis have become more prevalent with extended courses of therapy. The use of alternative complementary targets including epidermal growth factor receptor (EGFR) has been suggested; however, the mechanisms underlying EGFR and its potential role in chemoresistance to platinum‐based therapy are not fully understood. In this study, we elucidate the molecular signaling downstream of EGFR and its binding partner Prion protein (PrP^C^) that contribute to platinum resistance and development of metastases in patients.

The noninfectious cellular PrP^C^, encoded by the *PRNP* gene, is a protein of unknown precise function (Mehrpour and Codogno, 2010). PrP^C^ is involved in the progression of a number of cancers, including colorectal (Liang *et al*., 2006; Mehrpour and Codogno, 2010), breast (Diarra‐Mehrpour *et al*., 2004; Vassallo *et al*., 2005), gastric (Liang *et al*., 2006, 2007; Pan *et al*., 2006), and pancreatic (Li *et al*., 2009a), as well as potentially supporting drug resistance in gastric and breast cancers (Du *et al*., 2005; Li *et al*., 2009b). PrP^C^ is found mainly as a glycophosphatidylinositol‐anchored cell surface protein in lipid rafts. Association of PrP^C^ with signaling receptors in lipid rafts can either enhance or inhibit oncogenic signaling. The binding of EGFR by PrP^C^ and inhibition of subsequent signaling pathway have significant implications on cellular response to therapies. There is evidence that phosphatidylinositol 3‐kinase (Pi3K) signaling induces PrP^C^ expression by inhibition of the repressive action of the forkhead/winged helix box class O (FOXO) transcription factors on PrP^C^ expression (Liu *et al*., 2013). FOXO3a is a downstream effector of EGFR and the PI3K pathway, which via p38 mitogen‐activated protein kinase (MAPK) inhibition can induce cell cycle arrest and is a key mediator of the cytotoxic effect of cisplatin in colorectal cancer (Fernández de Mattos *et al*., 2008; Germani *et al*., 2014). We speculate that PrP^C^ can influence cisplatin resistance in colorectal cancer via EGFR signaling to p38 MAPK and regulation of expression of FOXO3a. Further, we propose that Krüppel‐like factor 5 (KLF5), the downstream effector of this pathway, promotes cisplatin/oxaliplatin resistance supporting metastasis in colorectal cancer patients.

## 2 Materials and methods

### 2.1 Cell culture

HT29, SW620, and T84 cell lines (ATCC, Noble Park, Australia) were cultured at 37 °C with 5% CO_2_ in high‐glucose Dulbecco's modified Eagle's medium (Invitrogen) supplemented with 10% FBS (Invitrogen, Scoresby, Australia) and a 1% penicillin/streptomycin cocktail (Invitrogen).

### 2.2 Immunofluorescence

Immunofluorescence microscopy was performed as previously described (Ham *et al*., 2016). Coverslips were incubated overnight at 4 °C with primary antibodies specific to PrP^C^ (3F4 Millipore, Bayswater, Australia) or EGFR (Ab2 Sigma, Castle Hill, Australia) (1 : 500 with PBS), washed with ice‐cold PBS, and stained with secondary anti‐mouse fluorophore‐labeled antibodies (1 : 5000 Sigma). Coverslips were mounted with ProLong Gold Antifade Mountant with DAPI liquid mountant (Life Technologies, Scoresby, Australia). Images were taken on a Zeiss 780‐NLO confocal microscope (Carl Zeiss Microscopy, North Ryde, Australia) with 40x and 100x magnifications.

### 2.3 Immunoblotting and immunoprecipitation

Immunoblots were probed with anti‐PrP^C^ (Millipore), anti‐FOXO3a (Abcam, Melbourne, Australia), anti‐phospho‐FOXO3a (Abcam), anti‐AKT (Cell Signaling), anti‐phospho‐AKT (Cell Signaling, Arundel, Australia), anti‐p38 MAPK (Cell Signaling), anti‐phospho‐p38 MAPK (Cell Signaling), and anti‐KLF5 (R&D Systems, Noble Park, Australia). Histone H3 (Cell Signaling) or α‐tubulin (Sigma) was used as loading control. Membrane fraction of cell lysates (100 μg) were incubated in the presence of 2 μg of either anti‐PrP^C^ (Millipore), anti‐EGFR (Cell Signaling), or IgG isotype control antibody overnight at 4 °C with mixing. To each immunoprecipitation 60 μL of Protein A/G agarose bead slurry (Millipore) was added and incubated with mixing for 2 h at room temperature. The beads were spun down and washed five times with ice cold PBS. The final spin was resuspended in SDS/PAGE loading buffer and 5 μL loaded per well. Anti‐HSP70 was used as a loading control for 1% input.

### 2.4 Drug dose curves

IC50 calculated using dose–response curves with cisplatin (0, 3, 10, 30, 100, and 300 μm), LY2228820 (0, 1, 3, 10, 30, and 100 μm), and gefitinib (0, 0.01, 0.1, 1, 10, and 100 μm) and analyzed for effects on cell viability via MTS assay after 72 h (Sigma, Castle Hill, Australia). Absorbance of samples was measured at 500 nm, and the percentage of viable cells was calculated using prism software (GraphPad, San Diego, CA, USA).

### 2.5 Gene silencing

HT29 and SW620 cells were seeded at 1.5 × 10^5^ cells per 35 mm well and incubated overnight. The cells were then transfected with siRNA using Lipofectamine 2000 in OptiMEM for 6 h. siRNA used include ON‐TARGETplus human PrP^C^ (Dharmacon, Sydney, Australia), human KLF5 (Dharmacon), and universal negative control (Sigma‐Aldrich, Castle Hill, Australia), at a final concentration of 10 nm.

### 2.6 Quantitative real‐time polymerase chain reaction

RNA was isolated using RNeasy Mini Kit (Qiagen, Chadstone, Australia). cDNA was synthesized using RT2 First Strand Kit (Qiagen). cDNA was analyzed on LightCycler 480 (Roche, North Ryde, Australia) with validated primer sets (Table S1). Fold expression change was calculated against β‐actin and expressed as base‐two exponential increase in RNA levels ($2^{-\Delta \Delta C_{t}}$) ± SEM.

### 2.7 Immunohistochemistry

Sections (3–4 μm) were deparaffinized and blocked in BSA for 15 min, and then incubated in primary antibody (1 : 500 with PBS) overnight at 4 °C. Sections were washed and MACH 1 Universal HRP‐Polymer Detection (Biocare Medical, Redcliffe, Australia) applied for 30 min. Sections were washed, and signal was developed in Betazoid DAB (Biocare Medical) for 5 min, and then washed in gently running tap water for 5–10 min to remove excess chromogen. Sections were then lightly counterstained in Mayer's hematoxylin, and then dehydrated through ascending graded ethanol, cleared in xylene, and mounted using DPX (Sigma‐Aldrich). Staining was interpreted by a pathologist (CL), who scored PrP^C^, FOXO3a, and KLF5 staining based on three criteria—intensity (nil, weak, moderate, or strong), localization (nuclear or cytoplasmic), and percentage (divided into 5% intervals). Weak staining was defined as visible at 400× magnification, moderate staining as visible at 100× magnification, and strong staining as visible at 20× magnification. Nuclear staining and cytoplasmic staining were assessed separately. Staining percentage was estimated and rounded to the closest 5%. For example, a tumor may have a result of ‘weak cytoplasmic staining in 45% of cells’. These semiquantitative measurements were converted to a final staining percentage.

### 2.8 Statistics

Results are presented as mean ± SEM of replicate analyses and are either representative of or inclusive of at least three independent experiments. All statistical analyses were performed using two‐tailed Student's *t*‐tests in graphpad prism 7 software. In all figures, significant differences between specified pair of conditions, as judged by *t*‐test, are indicated using asterisks (*P <* *0.05; **P <* *0.005, ***P <* *0.001). IC50 doses were calculated by interpolation of the sigmoidal dose–response curves (graphpad prism 7.0 software).

### 2.9 Human tissue samples

Formalin‐fixed, paraffin‐embedded tissue samples from 11 patients who had undergone surgery for colorectal cancer and matched liver metastasis between 2003 and 2014 Hokkaido University Hospital were utilized in this study. Clinicopathological information is summarized in Table 1. Formalin‐fixed, paraffin‐embedded tissue samples from 30 patients that underwent surgery between 2011 and 2012, and their MSI statuses were evaluated for BRAF^V600E^ mutation was screened on DNA extracted by the Chelex‐100 method (Bio‐Rad Laboratories, Hercules, CA, USA) using an allelic discrimination assay and KRAS mutation was assessed using High Resolution Melt technology (Whitehall *et al*., 2009).

**Table 1:** Changes in PrPC‐FOXO3a‐KLF5 expression in metastases determine patient outcome

Patient	T/N scoring	Stage at diagnosis	Adjuvant chemotherapy	Chemotherapy for metastasis	Outcome (month)	PrPC change colon vs Met (%)	Foxo change colon vs Met (%)	KLF5 change colon vs Met (%)
1	T4a/N2	IV	NONE	mFOLFOX6	Dead (36)	−5	65	0
5	T3/N1	III	mFOLFOX6	FOLFIRI	Dead (42)	−1	60	−1
6	T3/N2	IV	NONE	mFOLFOX6	Dead (24)	−30	10	0
2	T3/N0	II	XELOX	XELOX	Dead (13)	25	−20	15
7	T3/N2	IV	mFOLFOX6	FOLFIRI	Dead (28)	0	−45	4
3	T3/N1	IV	NONE	PMC	Alive (138)	5	−15	0
4	T3/N2	IV	NONE	mFOLFOX6	Alive (14)	1	−20	9
8	T3/N0	IV	NONE	mFOLFOX6	Alive (12)	−1	−30	−9
9	T2/N0	II	NONE	TS1	Alive (96)	0	−10	−4
10	T3/N1	IV	NONE	UFT+LV	Alive (102)	5	0	−4
11	T3/N0	IV	UFT	NA	NA	5	−15	0

Light grey: poor patient outcome based on increased FOXO3a expression. Dark grey: poor patient outcome based on increased KLF5 expression.

### 2.10 Human gene expression data

Analysis was performed using RNA‐seq data of 452 colon patient samples obtained from the TCGA repository. In the survival analysis, no restrictions based on gender, race, stage, grade, or molecular subtype were applied. A database with Affymetrix gene chips was set up as described previously (PMID: 27849044). In this, the analysis on gene expression was performed on 1211 patient samples restricted to Grade 3 tumors. Cox proportional hazards survival analysis was performed using each cutoff between the lower and upper quartiles, and the best performing cutoff was used to draw a Kaplan–Meier plot. The utilized probes were 1956 (EGFR) and 5621 (PRNP) in the RNA‐seq dataset and 209212_s_at (KLF5) and 210655_s_at (FOXO3A) in the Affymetrix dataset.

### 2.11 Study approval

All patients in this study provided written informed consent. Approval was obtained from Hokkaido University Human Research Ethics committee (HREC 14‐005) and QIMRB Human Research Ethics committee (HREC P1239 and P1278). The study methodologies conformed to the standards set by the Declaration of Helsinki.

## 3 Results

### 3.1 PrP^C^ overexpression promotes cisplatin resistance

**Figure 1:**
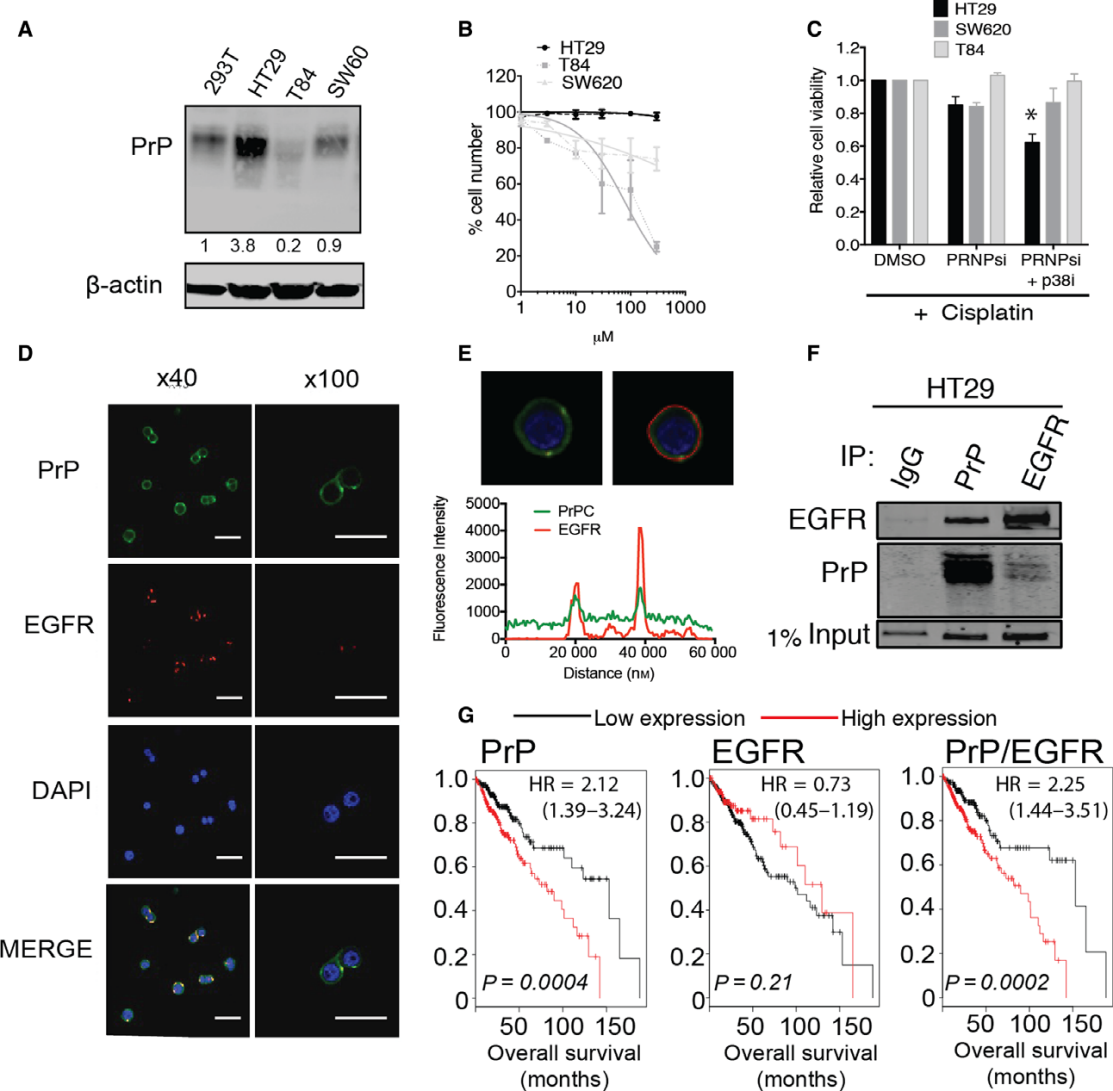
Prion protein associates with EGFR. (A) Western blot analysis of PrP^C^ expression in HT29, SW620, T84, and ‘normal‐like’ 293T cell lines. β‐Actin serves as a loading control. (B) Cisplatin dose–response curves of HT29, SW620, and T84 cell lines. Solid lines indicate normalized nonlinear fit and error bars ± SEM. (C) HT29, SW620, and T84 response to cisplatin treatment with or without PrP^C^ knockdown and p38 MAPK inhibitor. Cell viability was determined by MTS (% viability relative to DMSO control ± SEM and significance measured by two‐tailed Student's *t*‐test *P <* *0.05). (D) Subcellular localization of PrP^C^ (green), EGFR (red), and nuclei‐stained DAPI (blue), scale bar 100 μm. (E) Fluorescence signal intensity of PrP^C^ and EGFR in HT29 cells, indicating colocalization. (F) Immunoprecipitation of PrP and EGFR by reciprocal antibodies. HSP70 probed for 1% of lysate input. (G) RNA‐seq analysis of 452 colon patient samples for correlation between PrP^C^ and EGFR expression levels and patient outcome (significance determined by logrank test). All data are the means of three independent biological replicates and error bars ± SEM. PRNPsi, PrP^C^ knockdown; p38i, p38 MAPK inhibitor.

High PrP^C^ expression levels can promote chemoresistance and cancer progression by functions that include protein–protein interactions and transcriptional regulation (Cheng *et al*., 2014; Li *et al*., 2009b; Meslin *et al*., 2007; Zhao *et al*., 2002). Direct comparison of protein expression in colorectal cancer cell lines HT29, SW620, and T84 revealed 3.8‐fold overexpression of PrP^C^ in HT29 cells relative to the ‘normal‐like’ 293T cell line (Fig. 1A), which correlated with resistance to cisplatin (Fig. 1B). Cisplatin resistance in colorectal cancer is promoted by p38 MAPK signaling (Pereira *et al*., 2013). Inhibition of p38 MAPK has been shown to enhance the effects of cisplatin by activation of FOXO3a (Pereira *et al*., 2013). PrP^C^ promotes inhibition of FOXO3a activity to generate chemoresistance in neuroblastoma (Liu *et al*., 2013). We hypothesized PrP^C^‐mediated cisplatin resistance could be overcome by targeting p38 MAPK in a combination therapy with depletion of PrP^C^ expression. We previously determined the use of p38 MAPK inhibitor LY2228820 alone does not affect viability *in vitro* or affect tumor development of MDA‐MB‐231 breast cancer cells *in vivo* (Wiegmans *et al*., 2016). These cells express comparable levels of PrP^C^ to that observed in HT29 cells (Fig. S1A). Comparison of the colorectal cell lines revealed that only the low PrP^C^‐expressing T84 cell line was sensitive to LY2228820 (Fig. S1B). Of note, PrP^C^ knockdown alone (Fig. S1C) did not significantly overcome cisplatin resistance (Fig. 1C). However, addition of low‐dose p38 MAPK inhibitor significantly sensitized HT29 cells to cisplatin (Fig. 1C). This suggests that p38 MAPK is a major contributor to cisplatin resistance observed in the HT29 cell line. In colorectal cancer, p38 MAPK activation can be driven by EGFR (Grossi *et al*., 2014). We hypothesized that PrP^C^ supports EGFR activation and could be a marker for patient response to platinum therapy.

### 3.2 PrP^C^ and EGFR are associated in colorectal cells and are markers for patient outcome in colon cancer

Enhanced EGFR signaling in colorectal cancer promotes proliferation and cancer progression (Cohen, 2003; Salomon *et al*., 1995). PrP^C^ colocalizes with EGFR in lipid rafts and regulates EGFR function in neuronal cells (Llorens *et al*., 2013). We observed membrane expression of PrP^C^ and EGFR in HT29 cells (Fig. 1D), to a lesser extent in SW620 cells (Fig. S2A) and no detectable expression in T84 cells (data not shown). The two proteins colocalized at the cell membrane of HT29 cells when PrP^C^ was endogenously overexpressed (Fig. 1E), and each protein could be immunoprecipitated by the other in HT29 cells (Fig. 1F) and the smallest isoform of PrP^C^ by EGFR in SW620 cells (Fig. S2B). Examination of 452 colon patient samples for correlation between PrP^C^ and EGFR expression levels and patient outcome revealed that higher PrP^C^ expression is strongly associated with poor outcome, whereas EGFR was not (Fig. 1G). Coexpression of both PrP^C^ and EGFR was more significant than PrP^C^ alone and higher hazard ratio suggesting EGFR does contribute to poor patient outcome when PrP^C^ is overexpressed (Fig. 1G‐right panel).

### 3.3 Targeting p38 MAPK with depletion of PrP^C^ or targeting of EGFR overcome cisplatin resistance but through different signaling pathways

**Figure 2:**
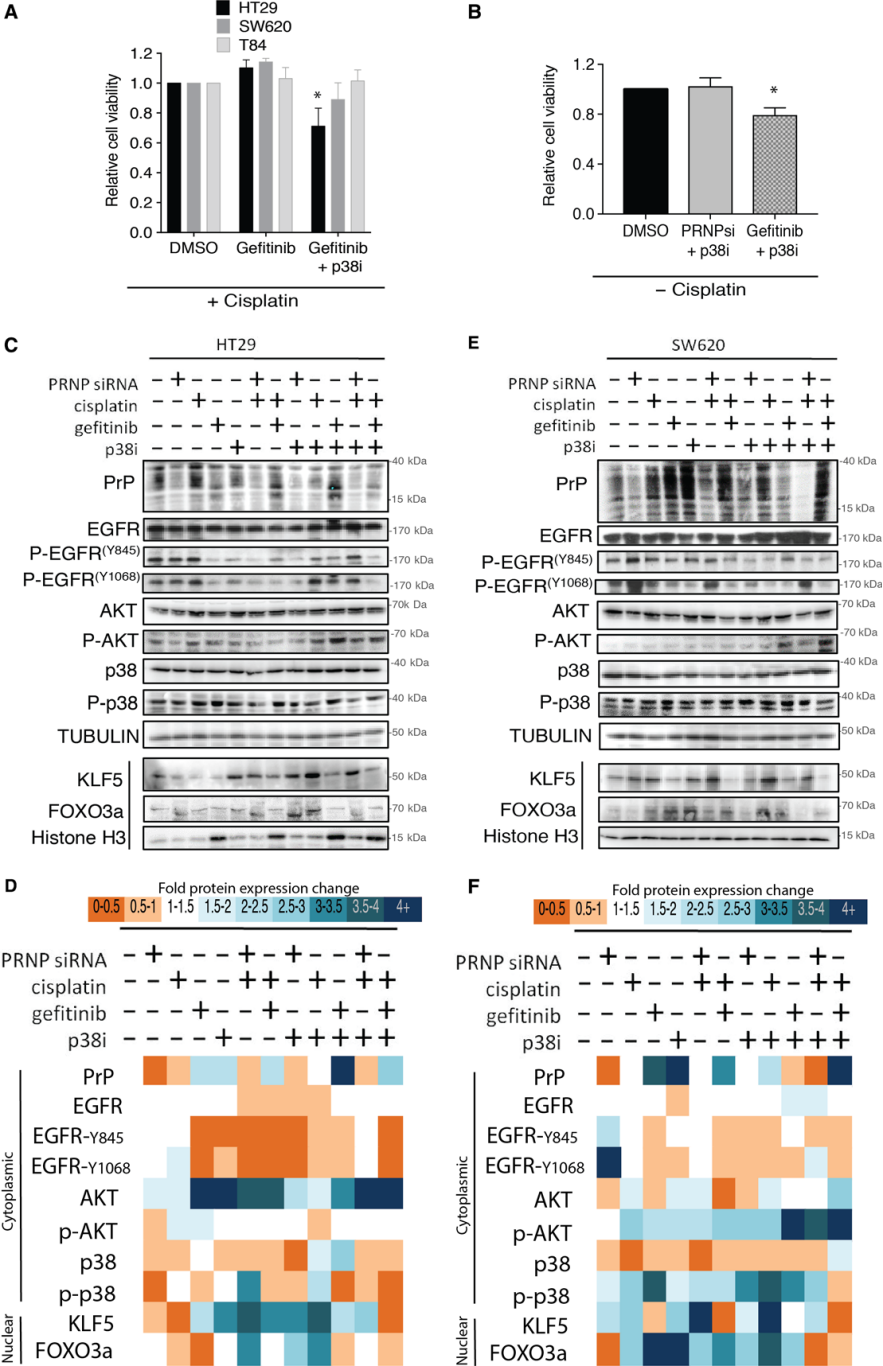
Analysis of signaling downstream of PrP^C^/EGFR by protein expression. (A) HT29, SW620, and T84 cell line viability in response to cisplatin treatment with or without gefitinib and p38 MAPK inhibitor. Cell viability determined by MTS (% viability relative to DMSO ± SEM and significance measured by two‐tailed Student's *t*‐test *P <* *0.05) (B) Cell viability of HT29 cells in response to p38 MAPK inhibitor with or without PrP^C^ knockdown and gefitinib in the absence of cisplatin. Cell viability determined by MTS (% relative to DMSO ± SEM and significance measured by two‐tailed Student's *t*‐test *P <* *0.05). (C) Protein expression analysis of the nuclear and cytosol fractions from HT29 cells from each treatment group. Histone H3 and α‐tubulin are used as loading controls for nuclear and cytosolic fractions, respectively. (D) Average densitometry of three independent isolates standardized to the controls and finally expressed as relative change to untreated cells. (E) Protein expression analysis of the nuclear and cytosol fractions from SW620 cells from each treatment group. Histone H3 and α‐tubulin are used as loading controls for nuclear and cytosolic fractions, respectively. (F) Average densitometry of two independent isolates standardized to the controls and finally expressed as relative change to untreated cells.

Inhibition of EGFR signaling with gefitinib results in growth delay of cancer cell lines expressing high levels of EGFR via activating FOXO3a by dephosphorylation, thus allowing its nuclear translocation (Krol *et al*., 2007). We investigated whether inhibition of EGFR would mimic PrP^C^ knockdown. The addition of gefitinib alone (Fig. S3) or in combination with cisplatin had no significant effect on any of the cell lines (Fig. 2A). Further addition of p38 MAPK inhibitor overcame cisplatin resistance in HT29 cells, similar to what was observed with PrP^C^ knockdown (Fig. 2A). We examined whether the contribution of p38 MAPK inhibition was the main driver of sensitivity for PrP^C^ knockdown or gefitinib and found that in the absence of cisplatin, PrP^C^ knockdown was ineffective with p38 inhibition, while gefitinib sensitized to p38 inhibition (Fig. 2B). We observed the same results with the dual EGFR/HER2 inhibitor afatinib (Fig. S4A,B). This indicates that PrP^C^ expression directly contributes to cisplatin resistance independent of EGFR signaling, which includes p38 MAPK.

To fully elucidate the mechanisms that support cisplatin resistance, we examined the signaling kinases downstream of EGFR in HT29 and SW620 cell lines. HT29 harbors *BRAF* ^V600E^ and *PI3K* ^P449T^ mutations, while SW620 harbors a *KRAS* ^*G12V*^ mutation all of which could provide independent constitutive signaling, affecting cisplatin response (Ahmed *et al*., 2013). The response of each of the cell lines to the drug combinations and or inhibitors was directly related to activation of the signaling pathway controlled by PrP^C^/EGFR and the nuclear levels of KLF5 protein. Western blot protein expression analysis and densitometry of independent experiments (Fig. S5) provided a heatmap of relative expression changes standardized for housekeeping protein expression. Generally, in the HT29 cells we observed changes in PrP^C^ expression under various conditions (Fig. 2C) and reduced EGFR and AKT signaling although AKT expression was enhanced across most conditions (Fig. 2D), indicating a dominant pathway via PI3K rather than BRAF. In contrast, P38MAPK expression was generally repressed. Interestingly, phoshpo‐p38 signaling was repressed by the combination of cisplatin/gefitinib but not by cisplatin/PrP^C^KD, indicating two different mechanisms of signaling controlled by EGFR and PrP^C^ in HT29 cells (Fig. 2D). A different overall profile was observed in SW620 cells with robust EGFR signaling across a number of conditions, of note in response to cisplatin and PrPC knockdown (Fig. 2E) Similarly, enhanced activation in response to cisplatin/gefitinib/p38i was seen with reduced EGFR signaling, KLF5, and FOXO3a protein levels in the nucleus (Fig. 2E). A more robust p38 signaling response via phospho‐p38 was observed in SW620 under various conditions, indicating a more dominant BRAF/p38 axis rather than PI3K (Fig. 2F). We evaluated whether targeting of the BRAF/p38 axis using a single dose of p38i could induce cell death over the extended period of 144 h of exposure. No significant cell death was observed even with sustained signaling response as indicated by induction of PrP^C^ protein expression (Fig. S6).

High expression levels of KLF5 are a determinant of resistance to cisplatin in ovarian and breast cancers (Dong *et al*., 2013; Li *et al*., 2017) and corresponded with enhanced EGFR signaling in both colorectal cell lines (Fig. 2C,E). The only combination therapy able to achieve low nuclear levels of KLF5 in both cell lines was cisplatin/gefitinib/p38i (Fig. 2D,F), which was also an effective cytotoxic combination (Fig. 2A). In HT29 cells, the absence of p38 inhibition in the cisplatin/gefitinib combination induced nuclear levels of KLF5, still demonstrating the requirement for targeting the p38 pathway and BRAF signaling even though it is not the dominant signaling pathway. In SW620 cells, in the absence of p38 inhibition the cisplatin/gefitinib combination repressed nuclear levels of KLF5, indicating the targeting of p38 may not be required in this setting (Fig. 2F).

### 3.4 Knockdown of KLF5 sensitizes to cisplatin more effectively than knockdown of PrP^C^

**Figure 3:**
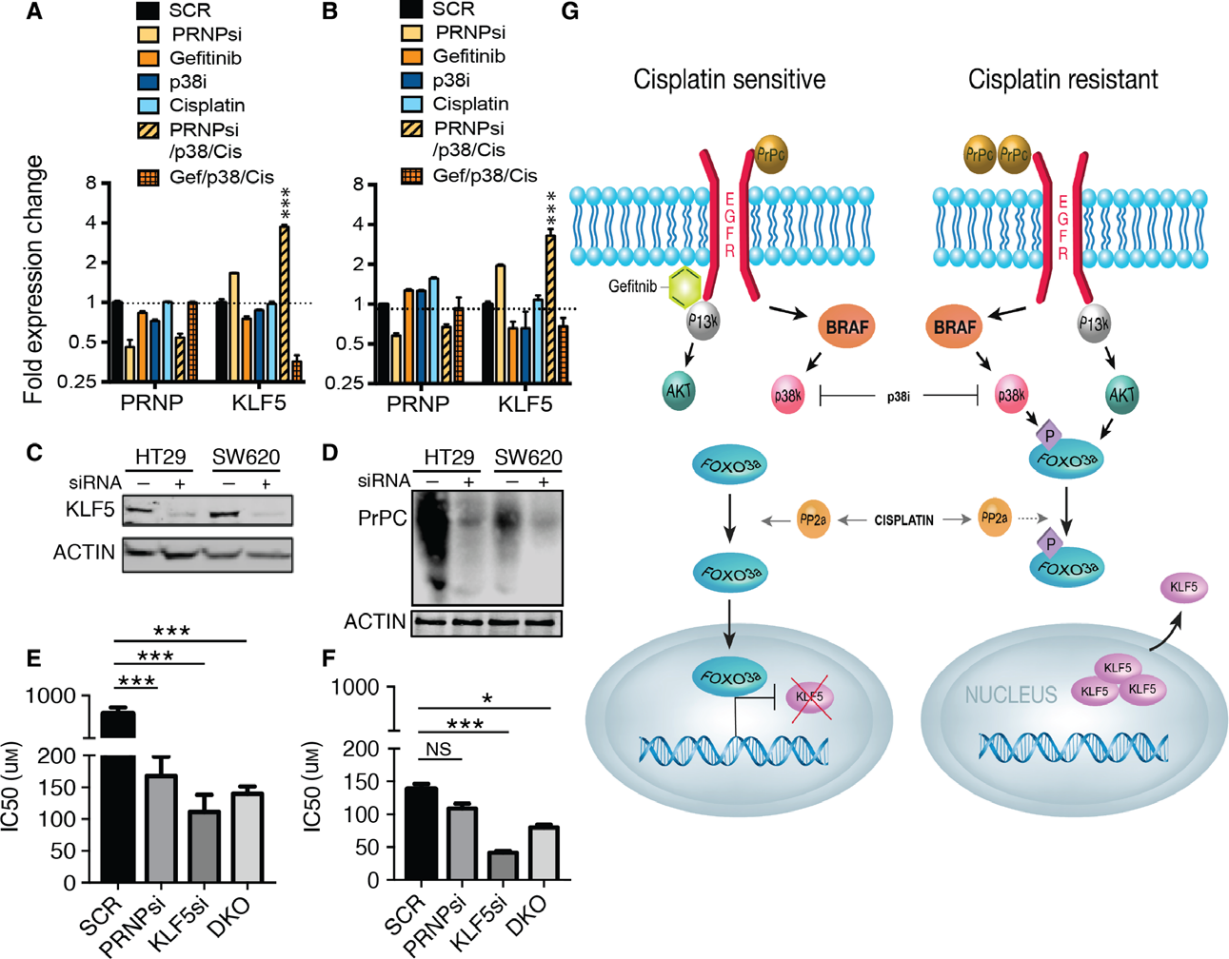
Knockdown of KLF5 sensitizes cells to cisplatin. (A) Relative mRNA expression levels of *PRNP* and *KLF5* in response to indicated treatment HT29 cells and (B) SW620 cells. (Data normalized to *GAPDH *± SEM and significance measured by two‐tailed Student's *t*‐test ***P <* *0.001) (C) Targeted knockdown of KLF5 in HT29 and SW620 cells. (D) Targeted knockdown of PrP^C^ in HT29 and SW620 cells. (E) Cisplatin IC50 in HT29 cells and (F) SW620 cells following PrP^C^, KLF5, or double knockdown determined by MTS. (Data compared to SCR control* *± SEM and significance measured by two‐tailed Student's *t*‐test ***P <* *0.001, *P < 0.05) SCR, nontargeting scrambled; PRNPsi, PrP^C^ knockdown; KLF5si, KLF5 knockdown; DKO, double (PrP^C^ and KLF5) knockdown. (G) A schematic showing the proposed EGFR signaling cascade in cisplatin‐sensitive and cisplatin‐resistant cells. The point of action of cisplatin, gefitinib, and p38 MAPK inhibitor is indicated.

We confirmed significantly decreased cDNA gene expression of KLF5 with the combination of cisplatin/gefitinib/p38i and significantly increased gene expression of KLF5 in response to cisplatin/PrP^C^KD/p38i in both cell lines (Fig. 3A,B). KLF5 typically supports proliferation in nontransformed cells, providing a growth advantage (Bateman *et al*., 2004; Chanchevalap *et al*., 2004; Sun *et al*., 2001), and has been shown to induce cisplatin resistance in breast cancer (Li *et al*., 2017). In contrast to these findings, KLF5 has been shown to have growth inhibitory properties in colon cancer‐derived cells (Bateman *et al*., 2004). We found that knockdown of KLF5 (Fig. 3C) sensitized to cisplatin to a greater extent than PrP^C^ knockdown alone (Fig. 3D) or knockdown of PrP^C^ and KLF5 together in HT29 cells (Fig. 3E) and SW620 cells (Fig. 3F). We suggest that resistance to platinum‐based therapy could be determined by the interplay between PrP^C^, EGFR, and resulting signaling to increase KLF5 expression levels and nuclear localization (Fig. 3G). We find that constitutively activating mutations result in a dominant signaling pathway but are not necessarily the pathway to be targeted to resensitize to the drug of choice, in this case cisplatin.

### 3.5 Activation of PrP^C^/FOXO/KLF5 axis correlates with platinum resistance and poor outcome in patients

**Figure 4:**
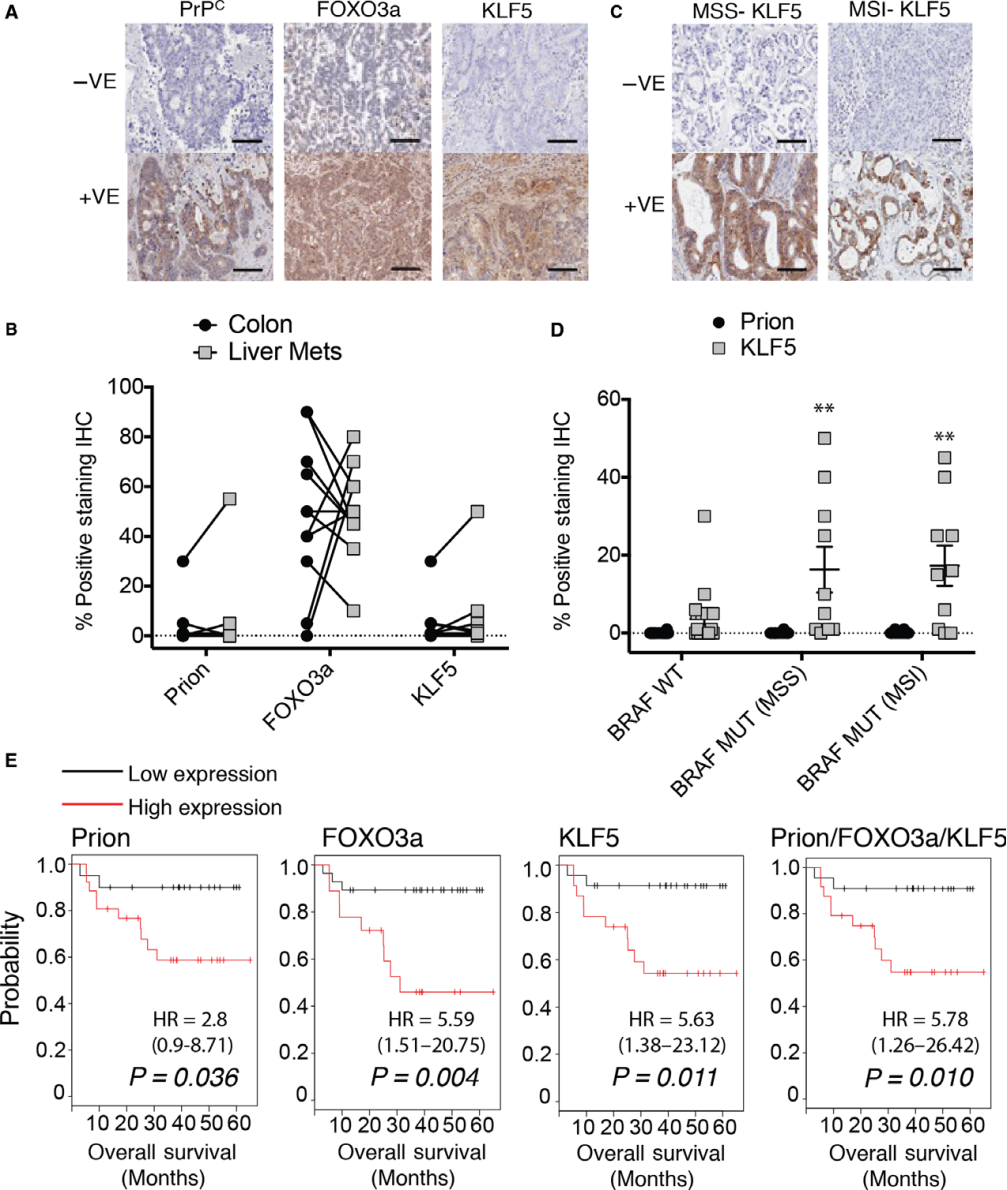
The PrP^C^/FOXO3a/KLF5 axis expression correlates with colorectal cancer patient progression and is a marker for outcome. (A) Representative images of negative and positive immunohistochemical staining of PrP^C^, FOXO3a, and KLF5 from liver metastases, scale bar 100 μm. (B) Changes in positive staining percentages for PrP^C^, FOXO3a, and KLF5 in 11 matched primary colorectal cancer and liver metastases. (C) Representative images of negative and positive KLF5 staining of *BRAF*‐mutant microsatellite stable (MSS) and *BRAF*‐mutant microsatellite unstable (MSI) cancers, scale bar 100 μm. (D) Comparison of PrP^C^ and KLF5 in *BRAF*‐wild‐type and *BRAF*‐mutant cancers, stratified by microsatellite instability (significance measured by two‐tailed Student's *t*‐test **P <* *0.005). (E) Gene expression correlation with overall survival in 46 patients with aggressive Grade 3 colon cancer (significance determined by logrank test).

We next examined changes in PrP^C^, FOXO3a, and KLF5 expression levels during progression from primary colorectal cancer to liver metastasis in matched patient samples, to determine a signaling response to platinum‐based therapy. Immunohistochemistry of tumor samples displays a range of positive and negative staining in the cytoplasm for PrP^C^, FOXO3a, and KLF5 (Fig. 4A). When chemotherapy history was taken into account, metastatic samples from three of the 11 patients exhibited decreased PrP^C^, increased FOXO3a, and decreased or no change in KLF5 compared with primary samples (Fig. 4B), signifying a platinum‐sensitive profile in the metastases. These three patients received oxaliplatin as part of their adjuvant therapy, which resulted in a median survival of 34 months (Table 1). Of the remaining eight patients, two patients received oxaliplatin, and their metastases displayed a resistance profile of increased PrP^C^, decreased FOXO3a, and increased KLF5 expression, resulting in a poorer outcome than patients with a sensitive profile with median survival 21 months (Table 1). Of the last six patients, four did not receive platinum‐based therapy and two had only recently commenced therapy at the time of sample collection.

We find that expression of KLF5 is an important determinant of cellular response in BRAF‐mutant cell line HT29 and in patient outcomes for platinum‐based therapy. KLF5 is upregulated in response to mutant *KRAS*, resulting in increased rate of proliferation and anchorage‐independent growth (Nandan *et al*., 2008); however, a link between chemoresistance mediated by KLF5 expression and *BRAF* mutation is yet to be described. We examined a cohort of *BRAF*‐wild‐type cancers and *BRAF*‐mutant cancers, further stratified by microsatellite instability status (Fig. 4C), and found KLF5 to be significantly elevated in *BRAF*‐mutant cancers (Fig. 4D). We did not observe any significant difference in PrP^C^ expression.

To evaluate the potential of the PrPc/FOXO3a/KLF5 axis to be prognostic in aggressive colon cancer, we examined a cohort of 46 Grade 3 patients for survival rates based on expression. Each of the genes in the axis displayed significant stratification for poor patient survival when overexpressed (Fig. 4E). Of note, FOXO3a gene expression displayed the most significance, which was not bettered by the three‐gene signature; however, we observed some variability in FOXO3a protein expression in our patient samples and suggest that the three‐gene signature would be a more robust prognostic predictor than a single gene (Fig. 4E).

## 4 Discussion

Epidermal growth factor receptor is a potential target for metastatic colorectal cancer with safety, tolerability, and pharmacokinetics being explored in multiple clinical trials. Potential efficacy and clinical outcome are determined by cellular molecular characteristics, including EGFR binding partners, cellular genetic aberrations, and available signal transduction pathways. In neuronal cells, PrP^C^ is a binding partner of EGFR creating a multimeric complex that colocalizes in the lipid rafts, which can be immunoprecipitated under endogenous levels of expression (Llorens *et al*., 2013). We observe colocalization in colorectal cancer cells. PrP^C^ has been shown to interact with two components of the EGFR macromolecular complex, Grb2 and p‐Src, revealing an active signaling complex that regulates both AKT and MAP kinase pathways. Upon depletion of PrP^C^, we observed reduced AKT signaling, signifying an important role for PrP^C^ in activation of EGFR signaling (Llorens *et al*., 2013). The consequences of reduced signaling resulted in reduced nuclear KLF5. KLF5 is present primarily in the epithelial cells lining the bases of the crypts and has been linked with cisplatin resistance in breast cancer (Li *et al*., 2017). This supports the hypothesis that PrP^C^ serves as a binding partner of EGFR and proto‐oncogene supporting colorectal cell proliferation and response to therapy via control of gene expression.

Chemosensitivity or chemoresistance is determined by the combination of genetic aberrations within the cancer cell that drive the dominant signaling. Recently, p38 MAPK and FOXO3a each have been described as potential factors in colorectal chemoresistance and possible drug targets (Grossi *et al*., 2014). The FOXO family of transcription factors are regulated by phosphorylation, ubiquitination, and/or acetylation, which affect subcellular localization and stability. As such, they are involved in a number of cellular processes including those observed to involve PrP^C^ (Brunet *et al*., 1999; van der Horst *et al*., 2006; Motta *et al*., 2004). FOXO3a has been demonstrated to be a key mediator of the cytotoxic effect of cisplatin (Fernández de Mattos *et al*., 2008; Germani *et al*., 2014). In cisplatin‐sensitive colorectal cancer cells, FOXO3a is dephosphorylated and undergoes nuclear translocation and target genes are expressed or repressed. However, this mechanism is compromised in those cell lines resistant to cisplatin (Fernández de Mattos *et al*., 2008). Of note, in colorectal cancer cells, signaling via p38MAPK represses FOXO3a activity and inhibition of p38MAPK has been shown to increase the effect of cisplatin by inducing FOXO3a dephosphorylation (Germani *et al*., 2014). In a neuroblastoma cancer model, there is evidence of PrP^C^ promoting chemoresistance by inhibition of FOXO3a (Liu *et al*., 2013). Therefore, we hypothesized that cisplatin resistance mediated by PrP^C^ could be overcome by targeting p38 MAPK. We observed PrP^C^ depletion alone did not sensitize HT29 or SW620 cells; however, in combination with p38 inhibition, we significantly sensitized HT29 cells to cisplatin. This suggests PrP^C^ and p38‐MAPK pathway are contributors to the cisplatin resistance observed in cell lines that express high levels of PrP^C^.

Mutations in either KRAS or BRAF drive colorectal cancers and predominate complementary pathways from EGFR that converge on FOXO3a. We found that mutations in upstream kinases such as KRAS or PI3K influenced the capability to activate p38MAPK downstream and there was an importance to target p38MAPK to sensitize to cisplatin with gefitinib in either cell line. However, overexpression of PrP^C^ induces p‐AKT promoting cell survival and chemoresistance (Llorens *et al*., 2013), which was enhanced in both colorectal cell lines by targeting p38 MAPK resulting in increased nuclear KLF5. This demonstrates that PrP^C^ may be signaling independent of EGFR. The targeting of p38 MAPK with additional depletion of PrP^C^ reduced p‐AKT and, however, retained nuclear KLF5 levels in both cell lines. This reveals that high nuclear KLF5 expression and the associated cisplatin resistance phenotype are driven predominantly by p38 MAPK signaling and not PrP^C^‐mediated induction of p‐AKT. This is supported by the loss of colorectal cell viability in the presence of gefitinib/p38i but not PRNPsi/p38i and reduced nuclear KLF5 expression with gefitinib/p38i/cisplatin but not PRNPsi/p38i/cisplatin. Of note, the HT29 colorectal cell line studied harbors a *PI3K* ^P449T^ mutation, which is likely to support AKT signaling to some extent independent of PrP^C^ status. PI3K mutations have been shown to provide enhanced sensitivity to gefitinib, with the isogenic breast cancer cell lines harboring either *PI3K* ^H1047R^ or *PI3K* ^E545K^ mutations 3.5 and 6.5 times more sensitive respectively to gefitinib than wild‐type cells (Glaysher *et al*., 2014). PI3K status has been identified as a key factor for response to anti‐EGFR treatment in metastatic colorectal cancer; however, we suggest that PrP^C^/EGFR may have utility in p38MAPK driven and BRAF colorectal cancers (Lièvre *et al*., 2017).

The prognostic value of PrP^C^/EGFR is related to analyzing the changes in expression of the PrP^C^‐FOXO3a‐KLF5 axis. As a gene set, the prognostic profile is very similar to that of KLF5 alone, but with slightly more statistical power. We found that patient outcomes were more favorable with repression of this axis rather than activation, resulting in a difference in median survival of 13 months. This is a meaningful difference as the current clinical outcomes based on nonprognosticated therapy are incremental at best. For example, the PRIME trial of 656 patients comparing the standard platinum‐based FOLFOX therapy with and without the targeting of EGFR signaling cascade demonstrated that targeting EGFR resulted in a significantly enhanced primary survival of 1.6 months and enhanced secondary survival of 4.2 months (Haraldsdottir and Bekaii‐Saab, 2013). If patients on this trial were stratified for cancers that were dominant for signaling via the PrP^C^‐FOXO3a‐KLF5 axis they could be switched off FOLFOX therapy, resulting in high rates of survival. With such a high proportion (50–70%) of acquired clinical resistance to platinum‐based therapy in colorectal cancer, we propose our findings have potential utility in prognosis and the ability to help track potential chemoresistance and metastatic relapse.

## 5 Conclusion

There is a need to identify the correlative molecular and pathologic markers that can predict patient outcome and guide therapy. We believe the PrP^C^‐FOXO3a‐KLF5 axis represents a novel molecular predictor of cisplatin resistance and associated metastatic relapse in aggressive colorectal cancer.

## Author contributions

CJA, APW, and AM designed the experimental plan and coordinated the collaborations and data analyses. CJA and APW executed most of the experiments. FK and VW provided the colorectal patient samples and associated history. CL scored the pathology. SH carried out immunofluorescence staining and analysis. BG provided access and helped in analysis of patient colorectal gene expression vs patient survival. ALM and MQW supervised CJA.

## Conflicts of interest

The authors declare no conflict of interest.

## Supporting information


**Fig. S1.** Characterization of colorectal cell lines.
**Fig. S2.** Co‐localization of EGFR and PrP^C^ in SW620 cells.
**Fig. S3.** EGFR inhibitor dose curves.
**Fig. S4.** Sensitization to Cisplatin with Afatinib and p38i.
**Fig. S5.** Densitometry of 3 independent western blots.
**Fig. S6.** Signaling dynamics resulting in PrP^C^ expression.
**Table S1.** Primers for quantitative PCR.
