# Supplementary material for: EGFR and Prion protein promote signaling via FOXO3a‐KLF5 resulting in clinical resistance to platinum agents in colorectal cancer
Source: Mol Oncol. 2019 Feb 8;13(4):725–37. doi: 10.1002/1878-0261.12411 (PMC6441932; doi:10.1002/1878-0261.12411)
Supplement: Supplementary file 1 — Fig. S1. Characterization of colorectal cell lines. Fig. S2. Co‐localization of EGFR and PrPC in SW620 cells. Fig. S3. EGFR inhibitor dose curves. Fig. S4. Sensitization to Cisplatin with Afatinib and p38i. Fig. S5. Densitometry of 3 independent western blots. Fig. S6. Signaling dynamics resulting in PrPC expression. Table S1. Primers for quantitative PCR. [file MOL2-13-725-s001.docx]

**A.**

**B.**

**A.**

**C.**

**Supp. Figure 1: Characterization of colorectal cell lines**. A) Comparative analysis of high PrP^C^ levels in HT29 versus breast cancer cell lines MDA-MB-231. B) LY228820 p38 MAPKi dose response curve analysis of HT29, SW620, T84 cell lines. Lines indicate non-linear fit of three independent experiments +/-SEM. C) PRNP and SCR siRNA transfected cells were subjected to whole cell lysis and subsequent SDS-PAGE and western blot analysis to confirm depletion of PrP^C^ expression.


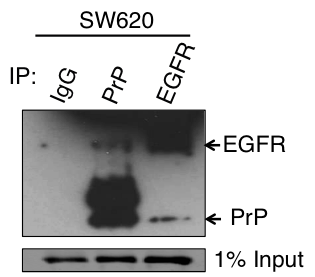


**B.**

**A.**

**Supp. Figure 2:** **Co-localization of EGFR and PrP^C^ in SW620 cells. A)** PrP^C^ (green) FOXO3a (red), and DAPI (blue), comparing PrP^C^ depleted and non-targeted cells. Images are x40 and x100 magnification. **B)** Immunoprecipitation of PrP and EGFR by reciprocal antibodies in SW620 cells. HSP70 probed for 1% of lysate input.

**A.**

**B.**

**Supp. Figure 3: EGFR inhibitor dose curves**. **A)** Gefitnib dose-response curve analysis of HT29, SW620, T84 cell lines. **B)** Afatinib dose-response curve analysis of HT29, SW620, T84 cell lines. Lines indicate non-linear fit of three independent experiments +/-SEM.

**B.**

**A.**

*

*

**Supp. Figure 4: Sensitization to Cisplatin with Afatinib and p38i. A)** HT29, SW620 and T84 response to cisplatin treatment with or without Afatinib and p38 MAPK inhibitor. **B)** Cell viability of HT29 cells in response to p38 MAPK inhibitor with or without PrP^C^ knockdown and Afatinib in the absence of cisplatin. Cell viability determined by MTS (% relative to DMSO, significance measured by two tailed student t-test**p*<0.05).


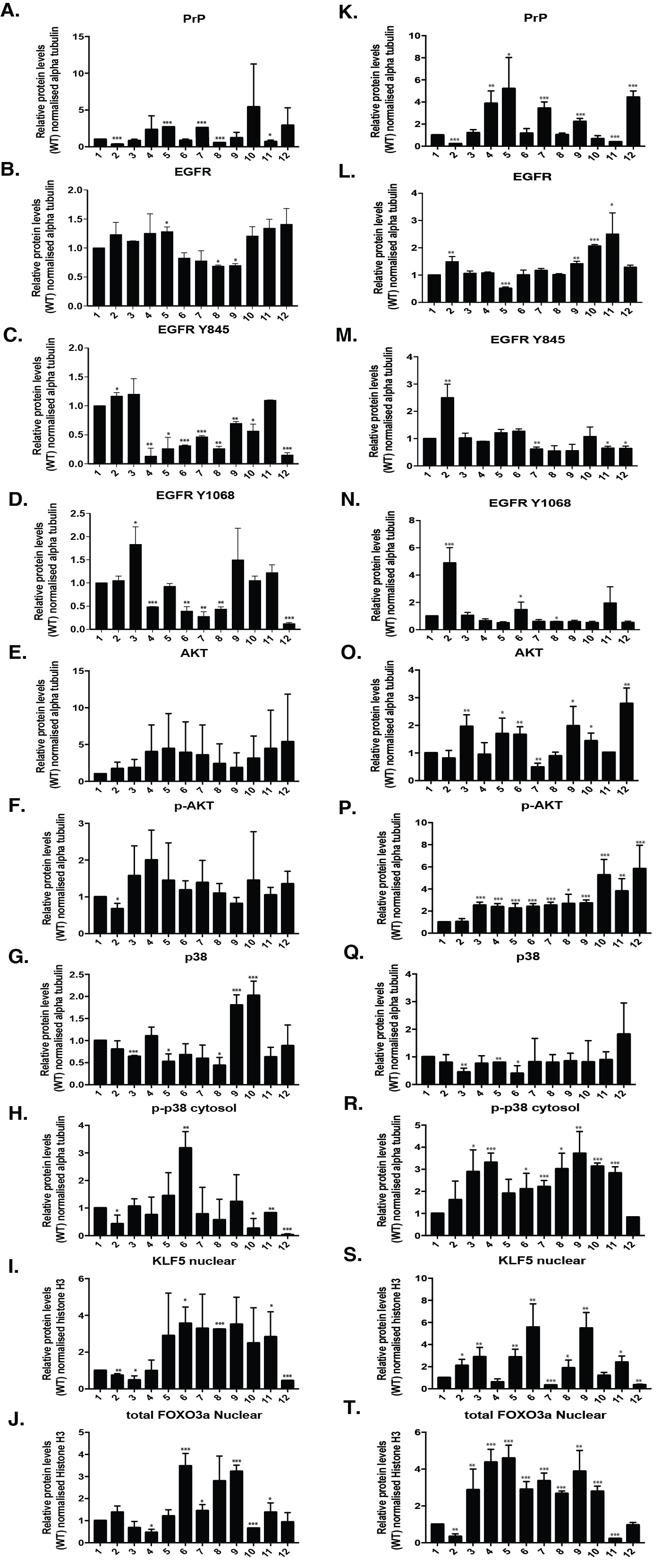


**KEY**

**1**- DMSO

**2**- PRNP siRNA

**3**- cisplatin

**4**- gefitinib

**5**- p38i

**6**- PRNPsi + cisplatin

**7**- cispltin + gefitinib

**8**- PRNPsi + p38i

**9**- cisplatin + p38i

**10**- gefitnib + p38i

**11**- PRNPsi + cisplatin + p38i

**12**- cisplatin + gefitinib + p38i

**Supp. Figure 5: Densitometry of 3 independent western blots**. (A-H) HT29 relative protein expression levels normalized to tubulin, (I-J) normalized to histone H3. (K-R) SW620 relative protein expression levels normalized to tubulin, (S-T) normalized to histone H3. All plots are average +/-SEM and two tailed student t-test for compared to DMSO treated controls for each condition **p<*0.01, ***p<*0.005 and ****p<*0.001.

**Supp. Figure 6: Signaling dynamics resulting in PrP^C^ expression**. Induction of PrP^C^ expression by p38 inhibition is sustained beyond 72 hours with a single dose of inhibitor without any significant sensitization of SW620 cells. Plot is average of 3 independent experiments +/-SEM.

| *PRNP* forward primer | TTCGGCAGTGACTATGAGGACC |
| --- | --- |
| *PRNP* reverse primer | TTGTGGTGACCGTGTGCTGCTT |
| *KLF5* forward primer | GAAGGAGTAACCCCGATTTGG |
| *KLF5* reverse primer | CTTCCCAGGTACACTTGTATGG |
| *RPLPO* forward primer | TGGTCATCCAGCAGGTGTTCGA |
| *RPLPO* reverse primer | ACAGACACTGGCAACATTGCGG |
| *GAPDH* forward primer | GTCTCCTCTGACTTCAACAGCG |
| *GAPDH* forward primer | ACCACCCTGTTGCTGTAGCCAA |

**Supplementary Table 1. Primers for quantitative PCR**
